# Crystal structure and Hirshfeld surface analysis of 2,5-dimethyl-1*H*-benzo[*d*]imidazole

**DOI:** 10.1107/S2056989026009072

**Published:** 2026-09-08

**Authors:** Achrafe Oulhaj, Jihad Sebhaoui, Gamal Al Ati, Fares Hezam Al-Ostoot, Joel T. Mague, El Mokhtar Essassi, Adam Duong, Karim Chkirate

**Affiliations:** a Laboratory of Heterocyclic Organic Chemistry URAC 21 Pharmacochemistry Competence Center Av Ibn Battouta BP 1014 Faculty of Sciences Mohammed V University in Rabat Morocco; b Laboratory of Glycochemistry and Agro-resources of Amiens, UR 7378, 10, Baudelocque Street, 80039 Amiens Cedex, University of Picardy Jules Verne, France; c Life and Health Sciences Laboratory, Faculty of Medicine and Pharmacy, Université Abdelmalek Essaâdi, Tangier, Morocco; d Department of Biochemistry, Faculty of Education & Science, Albaydha University, Albaydha, Yemen; e Department of Chemistry, Tulane University, New Orleans, LA 70118, USA; Katholieke Universiteit Leuven, Belgium

**Keywords:** crystal structure, hydrogen bond, C—H⋯π(ring) inter­actions, benzimidazole, Hirshfeld surface

## Abstract

The title mol­ecule, C_9_H_10_N_2_, exhibits whole-mol­ecule disorder in a 0.849 (4)/0.151 (4) ratio. The bicyclic portion is planar. In the crystal, a layer structure parallel to the *ac* plane is generated by N—H⋯N hydrogen bonds and C—H⋯π(ring) inter­actions. Hirshfeld surface analysis indicates that the most important contributions to the crystal packing are from H⋯H, C⋯H/H⋯C and N⋯H/H⋯N inter­actions.

## Abstract 2

The mol­ecule exhibits whole-mol­ecule disorder in a 0.849 (4)/0.151 (4) ratio. The bicyclic portion is planar. In the crystal, a layer structure is generated by N—H⋯N hydrogen bonds and C—H⋯π(ring) inter­actions.

## 1. Chemical context

Nitro­gen-based structures have attracted more attention in recent years due to their inter­esting properties in structural and inorganic chemistry (Al Ati *et al.* 2025 ▸; Ksama *et al.* 2025 ▸; Oufkir *et al.* 2026 ▸; Azgaou *et al.* 2026 ▸; Arzine *et al.* 2026 ▸). The family of benzimidazole derivatives is important in medicinal chemistry because of their wide range of pharmacological applications such as anti­bacterial (Chkirate *et al.*, 2020 ▸) and anti­oxidant (Chkirate *et al.*, 2023 ▸) activity. In particular, 2-methyl­benzimidazole is an anti-inflammatory and analgesic agent (Gaba *et al.*, 2010 ▸). Given the wide range of therapeutic applications for such compounds, and in continuation of the work already carried out for the synthesis of the compounds resulting from benzimidazole, a similar approach gave the title compound, 2,5-dimethyl-1*H*-benzo[*d*]imidazole C_9_H_10_N_2_ (I). Besides the synthesis, we also report the mol­ecular and crystal structures along with a Hirshfeld surface analysis.
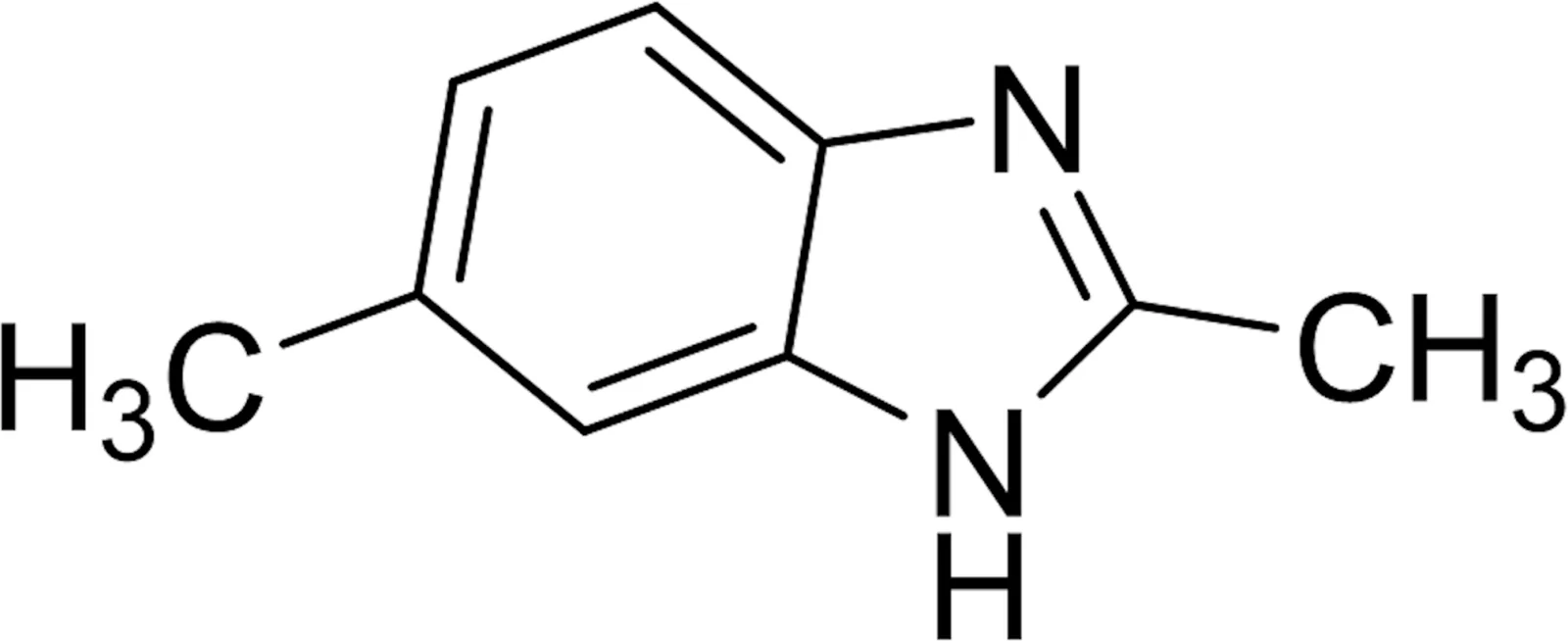


## 2. Structural commentary

**Figure 1:**
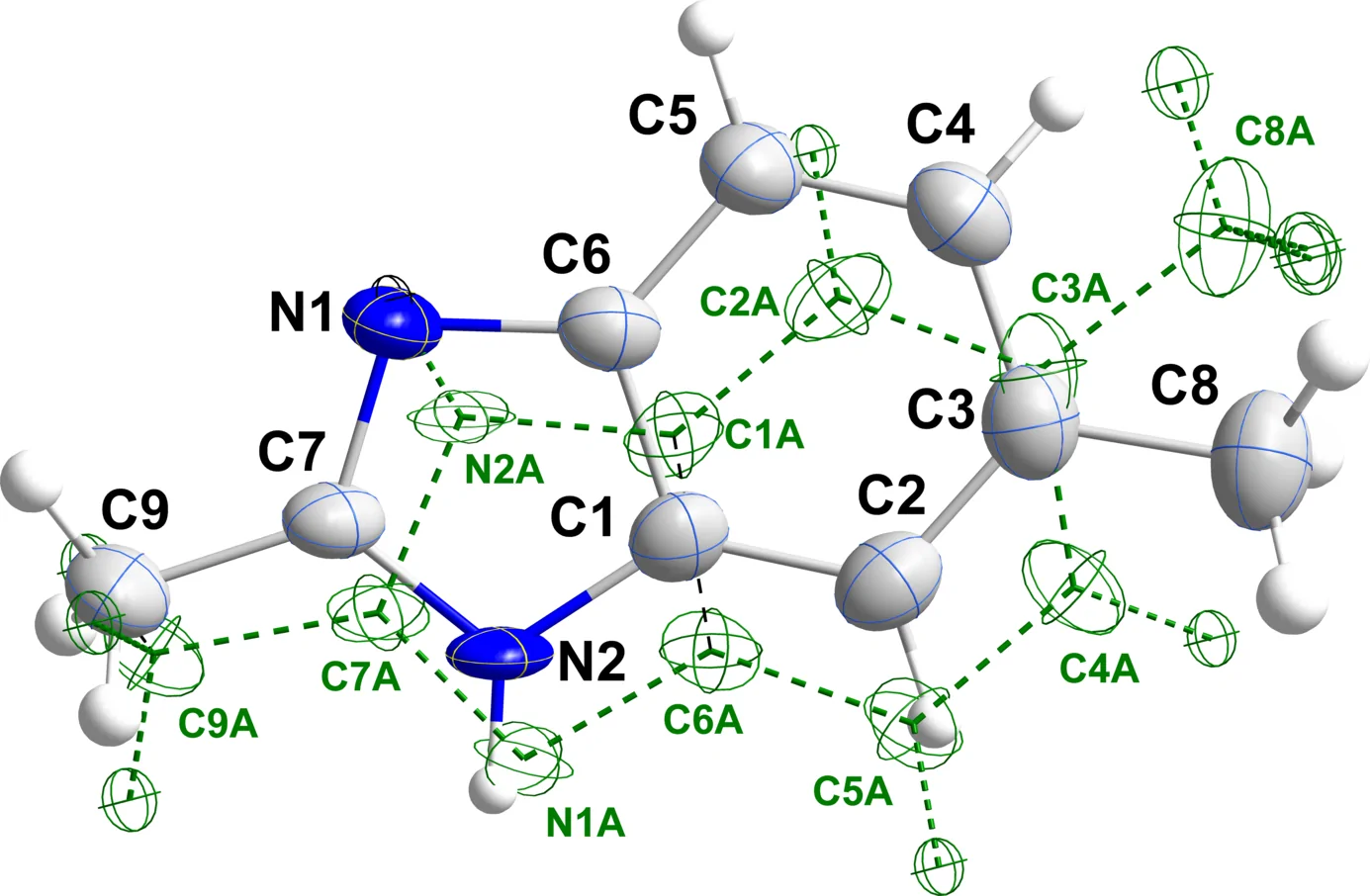
Perspective view of the title mol­ecule with labeling scheme and 50% probability ellipsoids. The minor component of the whole-mol­ecule disorder is depicted by the green skeleton and green labels.

There is whole-mol­ecule disorder in which the two orientations of the mol­ecule [refined ratio = 0.849 (4)/0.151 (4)] are related by a 180° rotation about the long axis and a translation of approximately 0.8 Å perpendicular to this axis in the plane of the mol­ecule (Fig. 1 ▸). The subsequent discussion refers to the major component of the disorder. The bicyclic portion of the mol­ecule is planar to within 0.012 (5) Å (r.m.s deviation of the nine fitted atoms is 0.0074 Å). Given the disorder, all bond lengths and bond angles appear as expected for the formulation given.

## 3. Supra­molecular features

**Figure 2:**
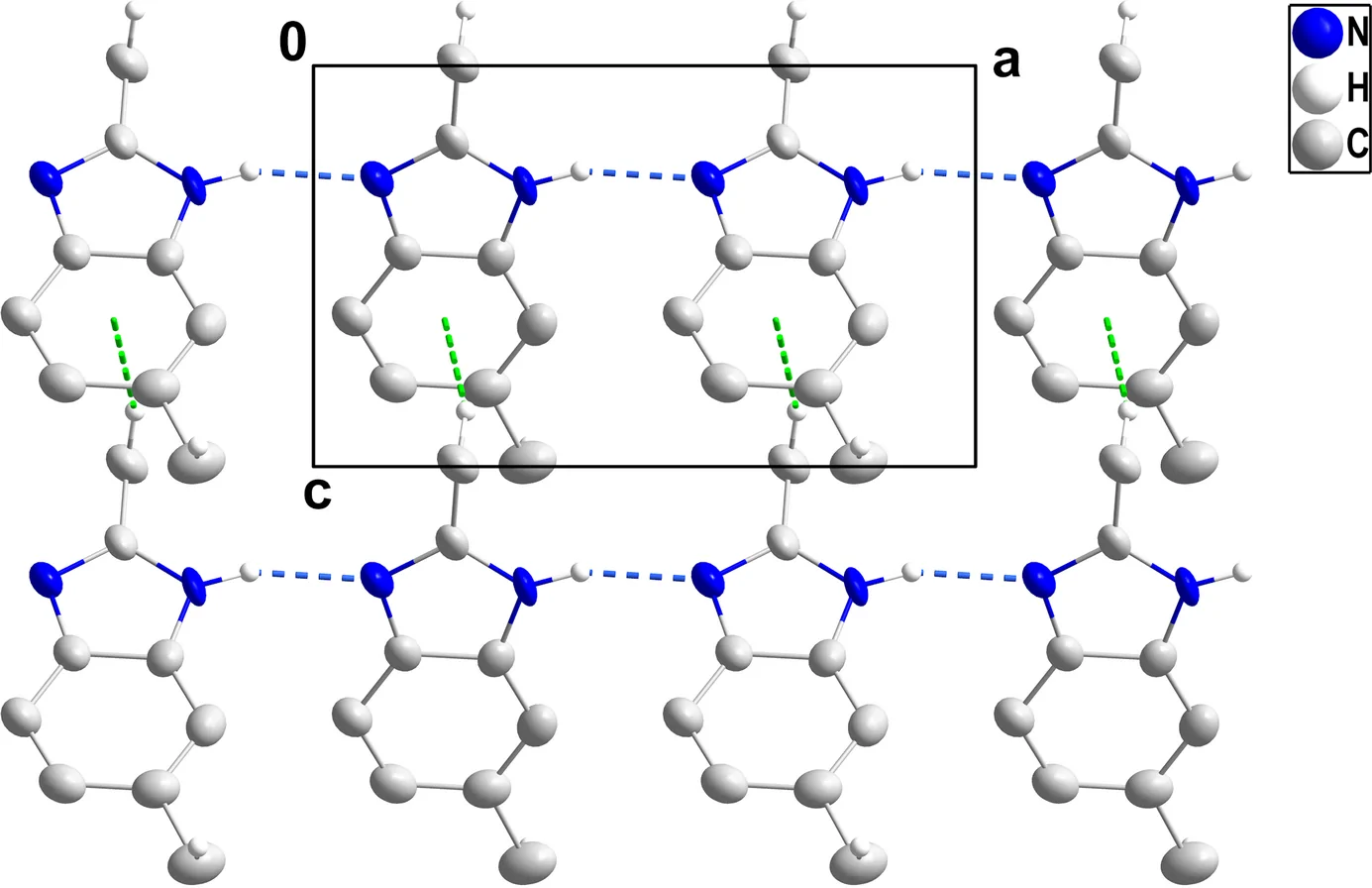
A portion of one layer of the major orientation viewed along the *b*-axis direction with N—H⋯N hydrogen bonds and C—H⋯π(ring) inter­actions depicted, respectively, by blue and green dashed lines. Hydrogen atoms not involved in these inter­actions are omitted for clarity.

**Figure 3:**
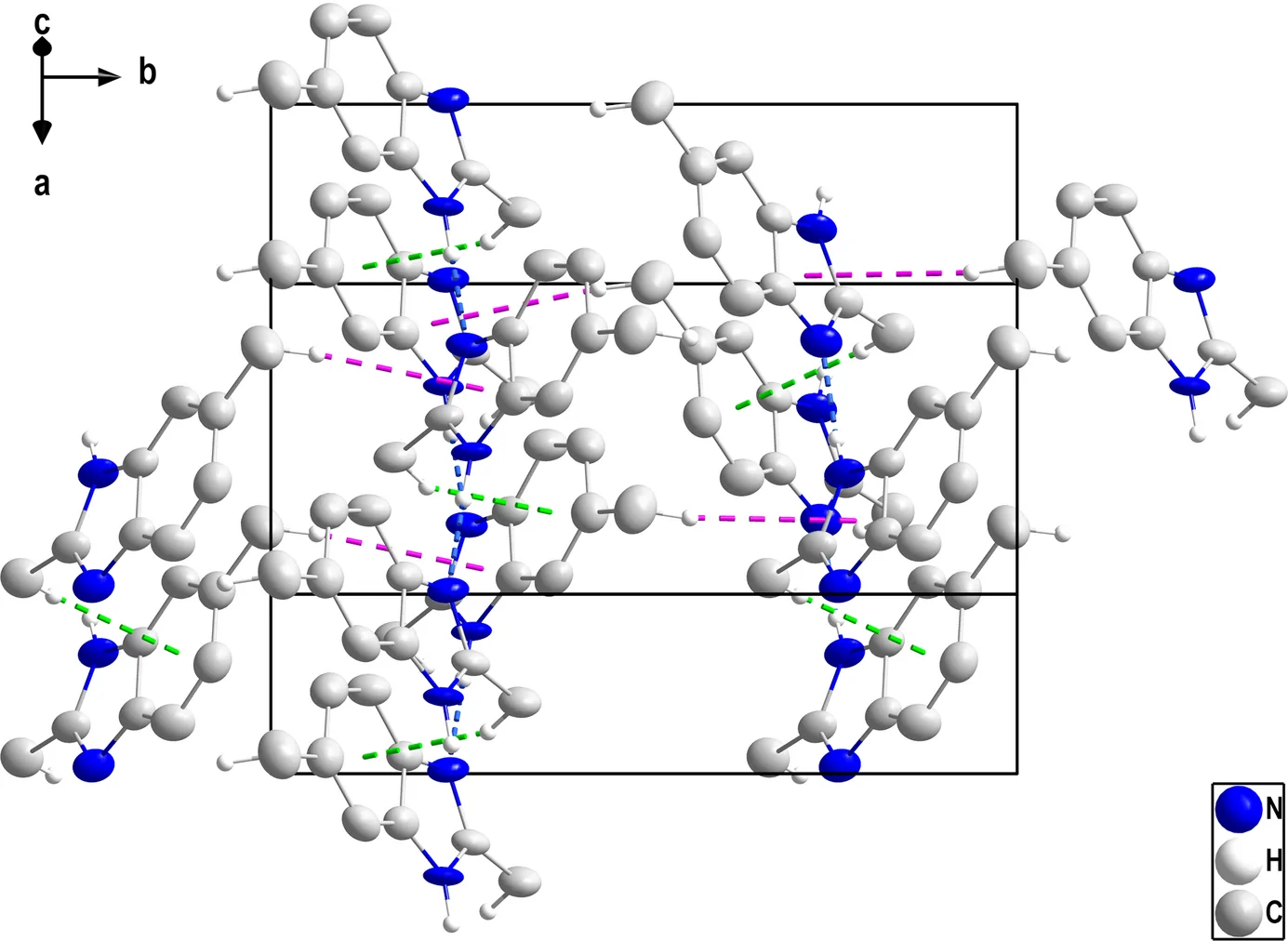
Packing of the major orientation projected on (101) with N—H⋯N hydrogen bonds depicted by blue dashed lines. The intra­layer C—H⋯π(ring) inter­actions are depicted by green dashed lines while those joining the layers are depicted by pink dashed lines. Hydrogen atoms not involved in these inter­actions are omitted for clarity.

In the crystal, N2—H2⋯N1^i^ hydrogen bonds form chains of mol­ecules extending along the *a*-axis direction, which are linked by C9—H9*B*⋯*Cg*2^iii^ inter­actions (Table 1 ▸) to form corrugated layers of mol­ecules parallel to the *ac* plane (Fig. 2 ▸). The layers are connected along the *b*-axis direction by C8—H8*C·*··*Cg*1^ii^ inter­actions (Table 1 ▸ and Fig. 3 ▸). There are no π–π stacking inter­actions present as confirmed by the Hirshfeld surface analysis showing C⋯C contacts to be only 0.8% of the total when only the major orientation was considered and only 0.6% of the total when both orientations were taken together.

## 4. Hirshfeld surface analysis

**Figure 4:**
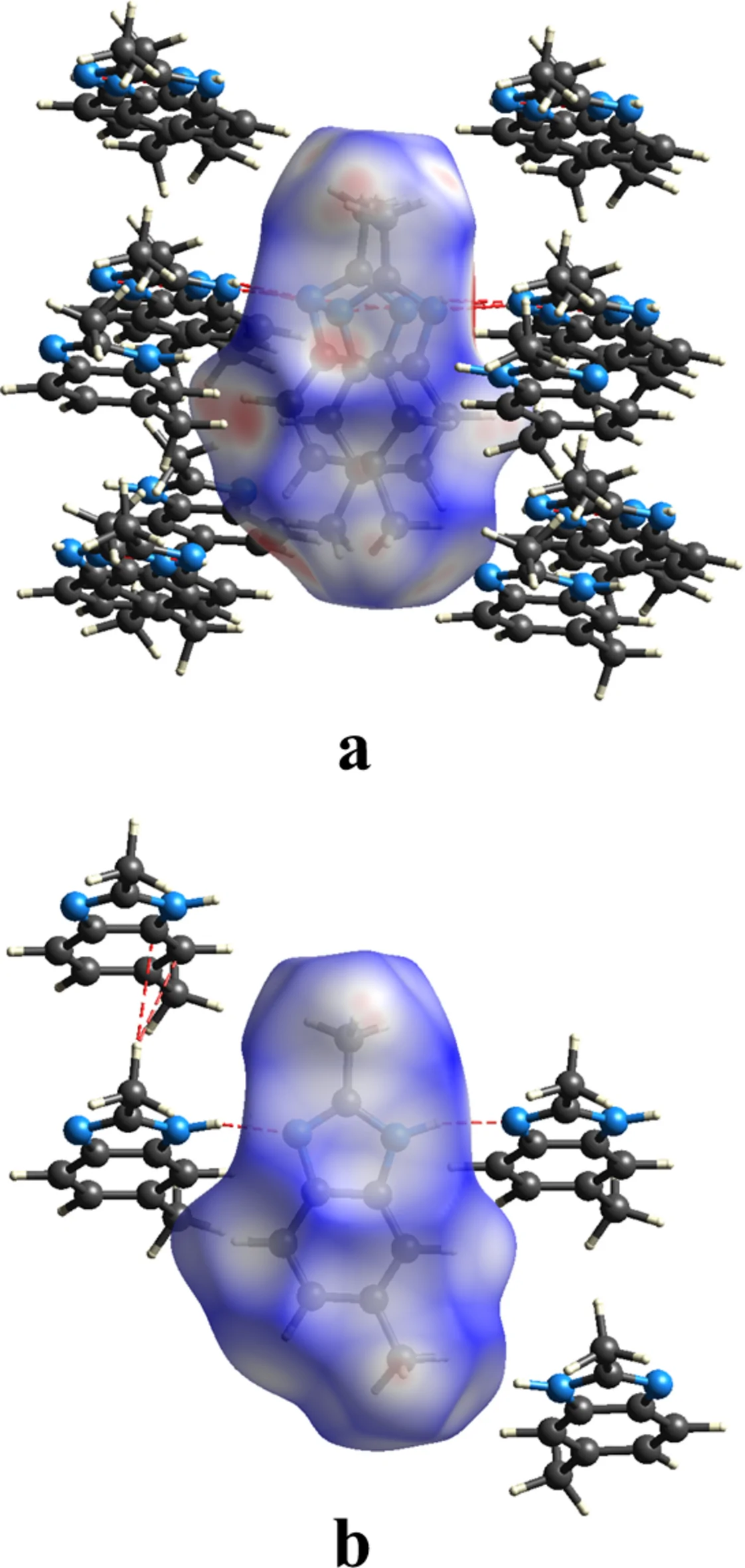
(*a*) View of the three-dimensional Hirshfeld surface of both orientations of the title compound taken together, plotted over *d*_norm_ in the range −0.6407 to 0.9694 a.u. (*b*) View of the three-dimensional Hirshfeld surface of the major orientation of the title compound plotted over *d*_norm_ in the range −0.6203 to 1.1594 a.u.

**Figure 5:**
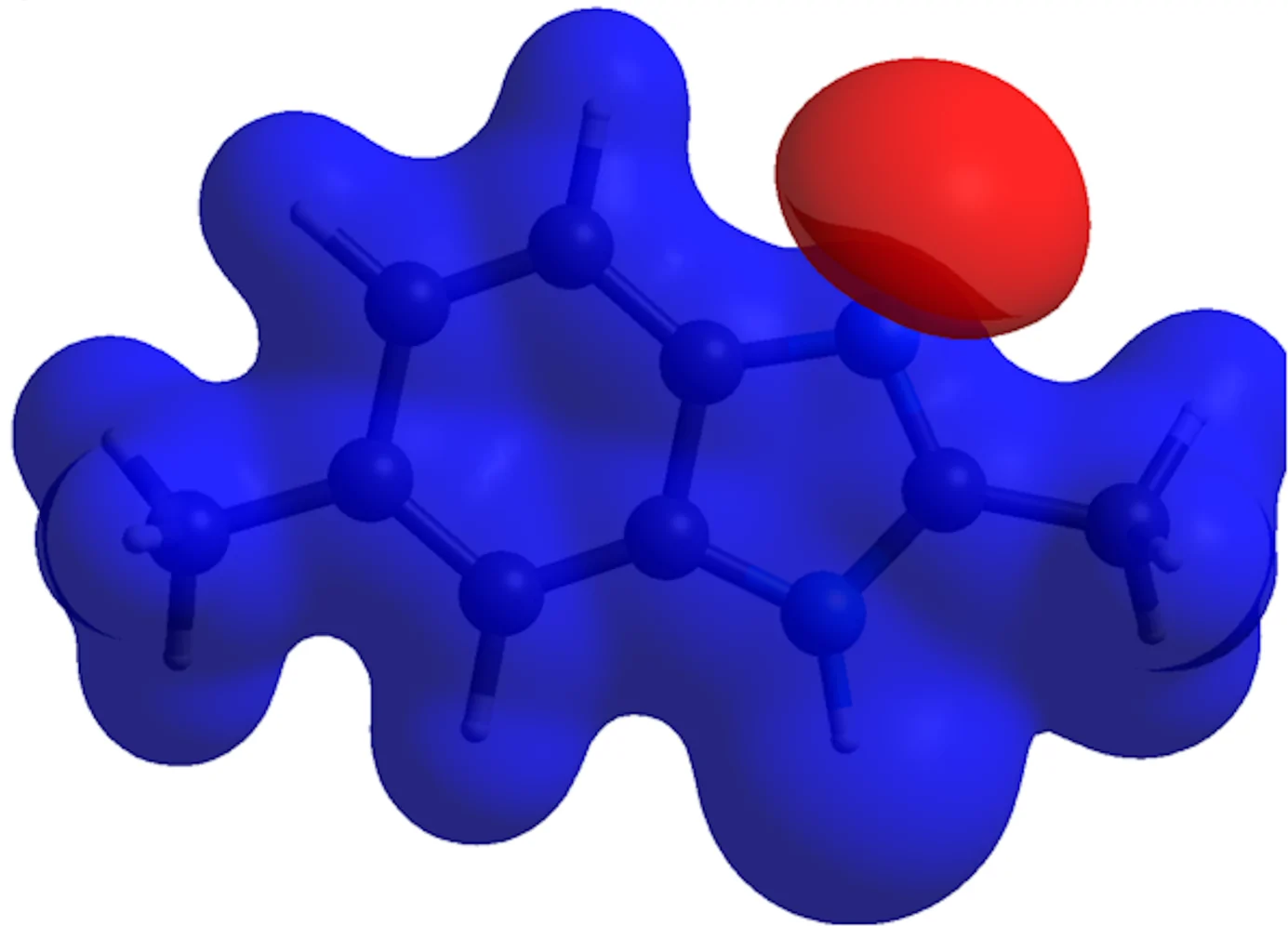
View of the three-dimensional Hirshfeld surface of the major orientation of the title compound plotted over the electrostatic potential in the range −0.0500 to 0.0500 a.u. using the STO-3G basis set at the Hartree–Fock level of theory.

The *CrystalExplorer* program (Turner *et al.*, 2017 ▸) was used to investigate and visualize the inter­molecular inter­actions of the title mol­ecule. The Hirshfeld surface for both orientations of the title mol­ecule plotted over *d*_norm_ in the range −0.6407 to 0.9694 a.u. is shown in Fig. 4 ▸*a* while that for the major orientation only and plotted over the range −0.6203 to 1.1594 a.u. is shown in Fig. 4 ▸*b*. In both instances, several neighboring mol­ecules are included. Only the N—H⋯N hydrogen bonds indicated in Fig. 4 ▸*a* by red dashed lines; in Fig. 4 ▸*b*, one of the C—H⋯π(ring) inter­actions is also depicted by two red dashed lines in the upper part of the figure. The electrostatic potential using the STO-3G basis set at the Hartree–Fock level of theory and mapped on the Hirshfeld surface for the major orientation over the range of ± 0.05 a.u. clearly shows the positions of close inter­molecular contacts in the compound (Fig. 5 ▸). The positive electrostatic potential (blue region) over the surface indicates hydrogen donor potential, whereas the hydrogen bond acceptors are represented by negative electrostatic potential (red region).

**Figure 6:**
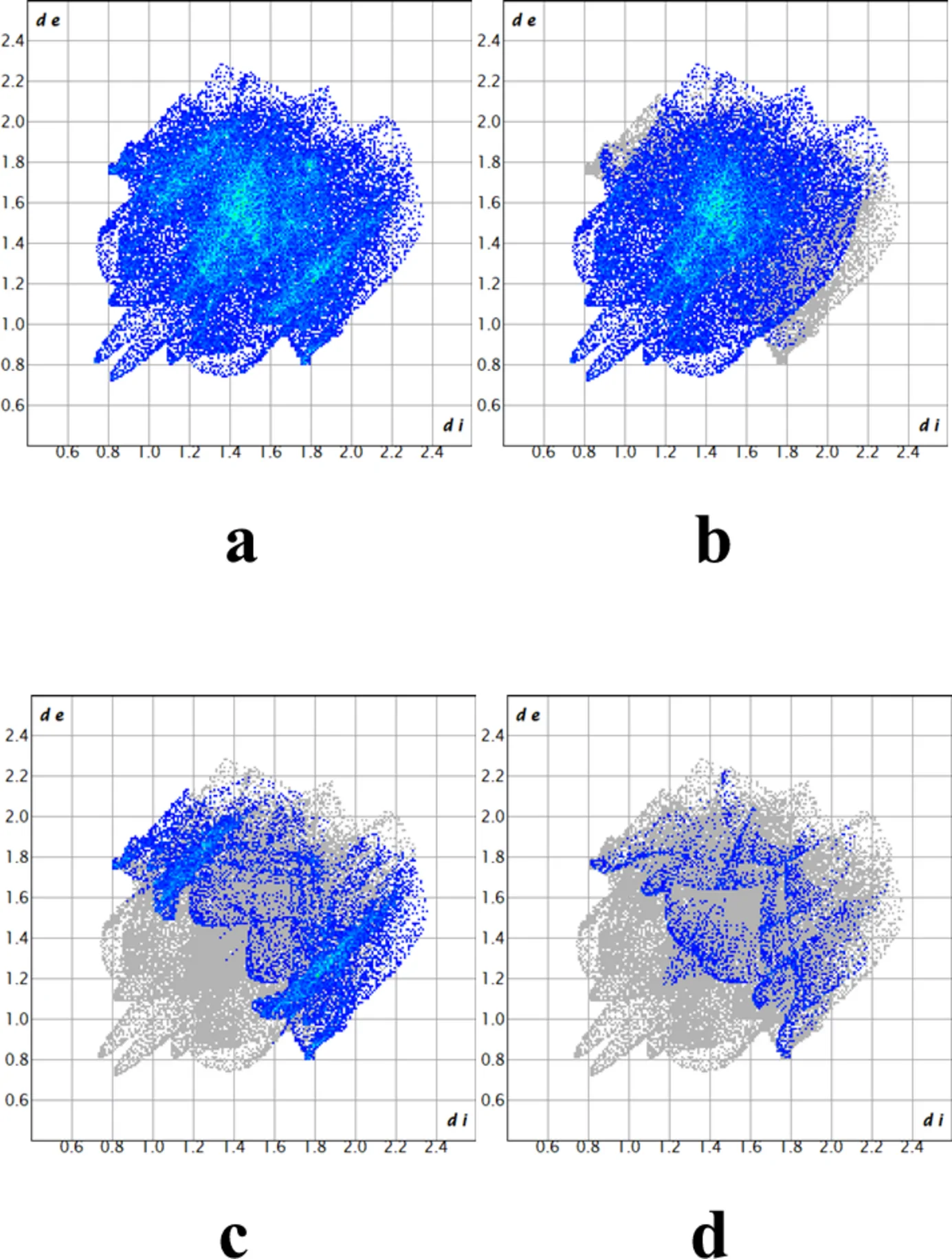
The full two-dimensional fingerprint plots for both orientations of the title compound taken together, showing (*a*) all inter­actions, and those delineated into (*b*) H⋯H, (*c*) C⋯H/H⋯C and (*d*) N⋯H/H⋯N inter­actions. The *d_i_* and *d_e_* values are the closest inter­nal and external distances (in Å) from given points on the Hirshfeld surface.

**Figure 7:**
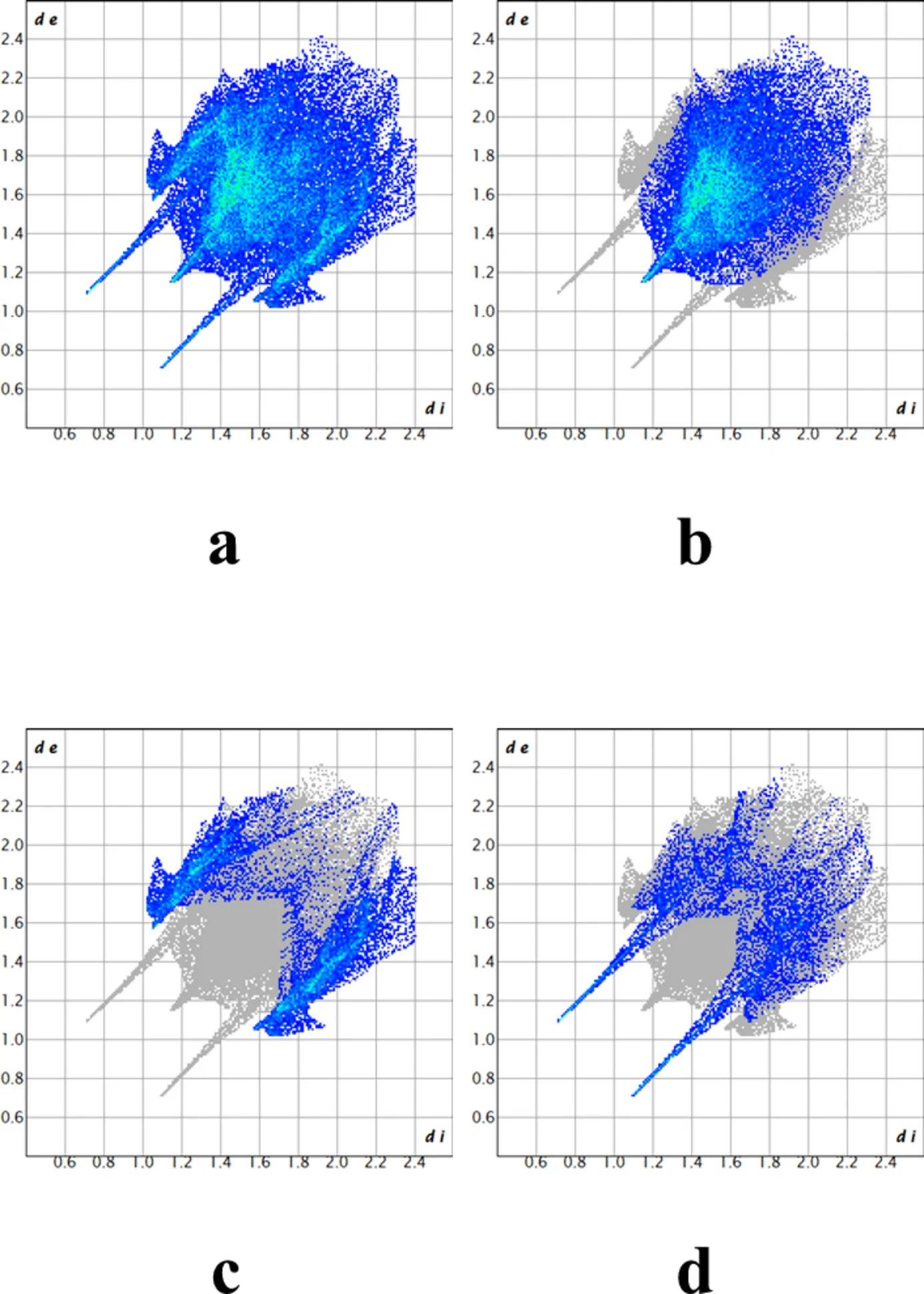
The full two-dimensional fingerprint plots for the major orientation of the title compound showing (*a*) all inter­actions, and those delineated into (*b*) H⋯H, (*c*) C⋯H/H⋯C and (*d*) N⋯H/H⋯N inter­actions. The *d_i_* and *d_e_* values are the closest inter­nal and external distances (in Å) from given points on the Hirshfeld surface.

The two-dimensional fingerprint plots (McKinnon *et al.*, 2007 ▸) for both orientations of the mol­ecule taken together are presented in Fig. 6 ▸, while the corresponding ones for the major orientation only are given in Fig. 7 ▸. All inter­molecular contacts are shown in Fig. 6 ▸*a* and 7*a* with those showing H⋯H contacts in Fig. 6 ▸*b* and 7*b*. The C⋯H/H⋯C contacts are given in Fig. 6 ▸*c* and 7*c* and the N⋯H/H⋯N contacts in Fig. 6 ▸*d* and 7*d*. In Fig. 6 ▸, the H⋯H, C⋯H/H⋯C and N⋯H/H⋯N contacts contribute 64.9%, 26.1% and 8.3% of the total inter­molecular inter­actions, respectively, while in Fig. 7 ▸ the contributions are 56.5%, 25.4% and 16.7%, respectively. The fact that the H⋯H contacts contribute the most is because the periphery of the mol­ecule consists largely of hydrogen atoms and, for both orientations, more of these contacts must be taken into account in the calculation of the surface area. Furthermore, because the atoms in the two orientations of the mol­ecule are located quite close together, there will be many small differences in the lengths of the inter­molecular contacts, which leads to rather diffuse graphs for the three specific inter­actions, as is clearly visible in Fig. 6 ▸*b*–6*d*. For the major orientation only, the majority of the C⋯H/H⋯C contacts (Fig. 7 ▸*c*) can be attributed to the C—H⋯π(ring) inter­actions while the pair of sharp peaks in Fig. 7 ▸*d* appearing at *d*_e_ + *d*_i_ ≃ 2.1 Å clearly represents the N—H⋯N hydrogen bonds.

## 5. Database survey

**Figure 8:**
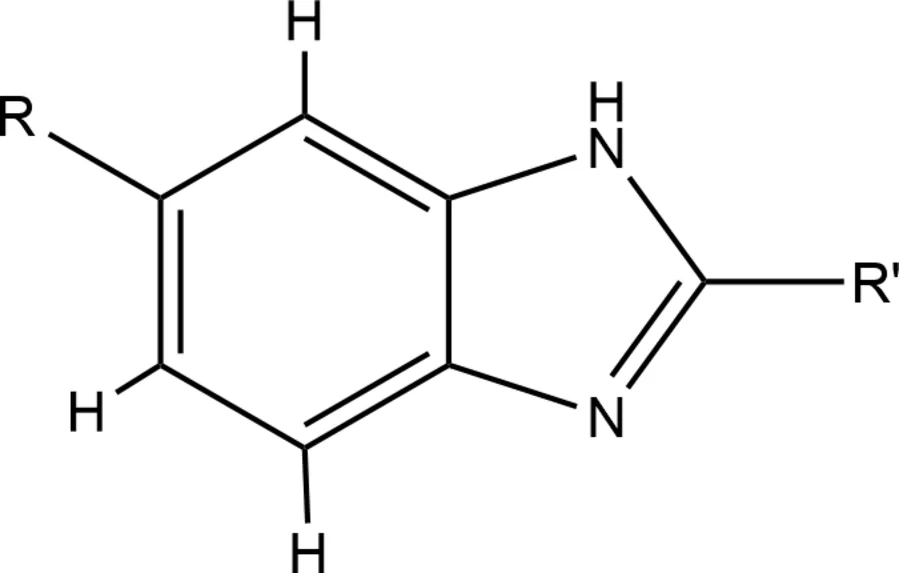
The search fragment used for the database survey.

A search of the Cambridge Structural Database (CSD version 6.01, updated May 2026; Groom *et al.*, 2016 ▸) with the 5-substituted-1*H*-benzimidazole fragment (Fig. 8 ▸, *R* = C, *R*′ = any atom or group) yielded 43 hits from which 27 examples were retained after elimination of protonated and polymeric structures as well as some duplicate entries. These are listed in Table 2 ▸. In most instances, the benzimidazole moiety is planar to within 0.02 Å, the only exceptions being AWIVEI, RIVWEB and XOWDOE where the maximum deviation of an atom in the bicyclic unit is 0.04, 0.03 and 0.04 Å, respectively. In MUSMOF, the benzimidazole moiety has crystallographically-imposed *m* symmetry. As in the title mol­ecule, most structures do not involve any π–πstacking inter­actions in the crystal. Exceptions are AGIVEU where neighboring five-membered rings show offset π-stacking, AWIVEI and YOTJUN where benzimidazole moieties π-stack across inversion centers, DUJLIE, TUBZEY and YOTJUN01 with the five-membered ring π-stacked with a neighboring pendant phenyl ring, TUBVOE where the five-membered ring inter­acts with the heterocyclic ring of a neighboring iso­quinoline unit, VUWGUT where the six-membered ring of the benzimidazole inter­acts with a pendant five-membered ring and ARUQUD which involves the π–π stacking of the five-membered ring of one benzimidazole with the six-membered ring of an adjacent one.

### 6. Synthesis and crystallization

A mixture of 3.36 g (0.02 mol) de­hydro­acetic acid and 4.88 g (0.04 mol) 4-methy-*o*-phenyl­enedi­amine in 80 mL of xylene was refluxed for two hours at 411 K. When the starting reagents had completely reacted, the solution was chromatographed on a silica gel column (chloro­form/ether: 60:40 *v*/*v*). The product was recrystallized from ethanol to give colorless, plate-like crystals of the title compound. ^1^H NMR (300 MHz, DMSO-*d_6_*) δ ppm: 2.44 (*s*, 3H, CH_3_ position 5) ; 2.70 (*s*, 3H, CH_3_ position 2); 7.12 (*d*, 1H, CH, *J* = 7.5Hz); 7.42 (*s*, 1H, NH); 7.43 (*s*, 1H, CH); 7.54 (*dd*, 1H, CH), *J* = 7.5Hz). ^13^C NMR (75 MHz, DMSO-*d*_6_) δ ppm: 14.0 (CH_3_); 21.3 (CH_3_); 115.1–125.8 (CH_arom_); 132.7–152.9 (C_q_).

## 7. Refinement

Crystal data, data collection and structure refinement details are summarized in Table 3 ▸. Following location of the minor orientation of the mol­ecule, the refinement was continued with the minor orientation restrained to have a comparable geometry to that of the major orientation using a SAME 0.002 N1 > C9 instruction. In addition, the displacement ellipsoids for the atoms of the minor component were linked to those of the major component with pair-wise EADP instructions while atoms N2, N2*A*, C7 and C7*A* were additionally restrained using ISOR 0.008. Hydrogen atoms were placed in idealized positions and included as riding contributions with isotropic displacement parameters tied to those of the attached atoms.

## Supplementary Material

Crystal structure: contains datablock(s) global, I. DOI: 10.1107/S2056989026009072/vm2334sup1.cif

Structure factors: contains datablock(s) I. DOI: 10.1107/S2056989026009072/vm2334Isup2.hkl

Supporting information file. DOI: 10.1107/S2056989026009072/vm2334Isup3.cml

CCDC reference: 2584534

Additional supporting information:  crystallographic information; 3D view; checkCIF report

## Figures and Tables

**Table 1:** Hydrogen-bond geometry (Å, °) *Cg*1 and *Cg*2 are the centroids of the N1/C6/C1/N2/C7 and C1–C6 rings, respectively.

*D*—H⋯*A*	*D*—H	H⋯*A*	*D*⋯*A*	*D*—H⋯*A*
N2—H2⋯N1^i^	0.88	1.95	2.801 (6)	162
C8—H8*C*⋯*Cg*1^ii^	0.98	2.97	3.941 (8)	172
C9—H9*B*⋯*Cg*2^iii^	0.98	2.57	3.478 (10)	153

Symmetry codes: (i) 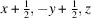; (ii) 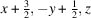; (iii) 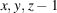.

**Table 2:** Results of database survey

REFCODE	*R*	*R*′	Reference
AGIVEU	COOEt	*p*-anis­yl	Yeong *et al.* (2018 ▸)
AWIVEI	morpholino­meth­yl	(3-cyclo­propyl­ureido)-1*H*-pyrazol-4-yl	Howard *et al.* (2009 ▸)
CINTIG	benzo­yl	2-hy­droxy-4-di­ethyl­amino­phen­yl	Bal *et al.* (2024 ▸)
DUJLIE	Me	2-hy­droxy-3-methyl­phen­yl	Eltayeb *et al.* (2009*a* ▸)
FIPJIZ01	(3,5-di­chloro-2-hy­droxy­phen­yl)-1*H*-benzo[*d*]imidazol-2-yl	3,5-di­chloro-2-hy­droxy­phen­yl	Geng *et al.* (2014 ▸)
HONLUT	5-methyl-7-nitro-1*H*-benzo[*d*]imidazol-2-yl	pyridin-2-yl	White *et al.* (2014 ▸)
JOHCUG	1,5-diisopropyl-6-oxo-1,2,5,6-tetra­hydro-1,2,4,5-tetra­zin-3-yl	H	Seber *et al.* (2014 ▸)
LOWNAP	morpholino­benzo[*d*]thia­zol-2-yl	pyridin-2-yl	White & Fellowes (2019 ▸)
MUSMOF	tri­fluoro­meth­yl	2-(5-(tri­fluoro­meth­yl)-1*H*-benzo[*d*]imidazol-2-yl)pyridin-6-yl	Fares *et al.* (2020 ▸)
OPUCUA	Me	(5-methyl­isoxazol-3-yl)meth­yl	Idrissi *et al.* (2021 ▸)
PIDJOD*^*a*^*	benzo­yl	NHCOOMe	Chen & Lu (2013 ▸)
PIDJUJ*^*b*^*	benzo­yl	NHCOOMe	Chen & Lu (2013 ▸)
PIDKAQ*^*c*^*	benzo­yl	NHCOOMe	Chen & Lu (2013 ▸)
PIDKEU*^*d*^*	benzo­yl	NHCOOMe	Chen & Lu (2013 ▸)
RALSUT	Me	{[3-bromo-1-(methyl­sulfon­yl)-1*H*-indol-2-yl]meth­yl}thio	Ravishankar *et al.* (2005 ▸)
RIFDAM	COOEt	H	Ding *et al.* (2005 ▸)
RIVWEB	(4-methyl­piperazin-1-yl)-1*H*-benzo[*d*]imidazol-2-yl	2-methyl-4-dimethylamino­phen­yl	White *et al.* (2018 ▸)
SAGQEW	benzo­yl	NHCOOMe	Caira *et al.* (1998 ▸)
TUBVOE	(4-methyl­piperazin-1-yl)-1*H*-benzo[*d*]imidazol-2-yl	isoquinolin-1-yl	White *et al.* (2019*a* ▸)
TUBZEY	(3-fluoro-6-morpholino-2-nitro­phen­yl)carbamo­yl	pyridin-2-yl	White *et al.* (2019*b* ▸)
VEVPOD	benzo­yl	NHCOOMe	Chen *et al.* (2012 ▸)
VUWGUT	1-(3,4,5-tri­meth­oxy­phen­yl)-1*H*-1,2,4-triazol-3-yl	H	Deb *et al.* (2024 ▸)
WOBKEF	(3,5-di­bromo-2-hy­droxy­phen­yl)-1*H*-benzo[*d*]imidazol-2-yl	3,5-di­bromo-2-hy­droxy­phen­yl	Geng *et al.* (2014 ▸)
XOWDOE	2-amino­pyridin-3-yl	(furan-2-ylmeth­yl)carbamo­yl	Jose *et al.* (2015 ▸)
YOTJUN	Me	2-hy­droxy-3-methyl­phen­yl	Xiao *et al.* (2009 ▸)
YOTJUN01	Me	2-hy­droxy-3-methyl­phen­yl	Eltayeb *et al.* (2009*b* ▸)
ARUQUD	Me	2-hy­droxy­phen­yl	Kubicki (2025 ▸)

Notes: (*a*) *n*-hexa­noic acid solvate; (*b*) *n*-penta­noic acid solvate; (*c*) *n*-butanoic acid solvate; (*d*) acetic acid solvate.

**Table 3:** Experimental details

Crystal data	
Chemical formula	C_9_H_10_N_2_
*M*_r_	146.19
Crystal system, space group	Orthorhombic, *Pna*2_1_
Temperature (K)	125
*a*, *b*, *c* (Å)	10.011 (3), 13.047 (4), 6.0600 (19)
*V* (Å^3^)	791.5 (4)
*Z*	4
Radiation type	Mo *K*α
μ (mm^−1^)	0.08
Crystal size (mm)	0.26 × 0.23 × 0.05

Data collection	
Diffractometer	Bruker D8 QUEST PHOTON 3 diffractometer
Absorption correction	Numerical (*SADABS*;Krause *et al.*, 2015 ▸)
*T*_min_, *T*_max_	0.98, 1.00
No. of measured, independent and observed [*I* > 2σ(*I*)] reflections	5699, 1599, 1106
*R*_int_	0.062
(sin θ/λ)_max_ (Å^−1^)	0.624

Refinement	
*R*[*F*^2^ > 2σ(*F*^2^)], *wR*(*F*^2^), *S*	0.069, 0.206, 1.09
No. of reflections	1599
No. of parameters	134
No. of restraints	54
H-atom treatment	H-atom parameters constrained
Δρ_max_, Δρ_min_ (e Å^−3^)	0.16, −0.31
Absolute structure	Flack *x* determined using 373 quotients [(*I*^+^)−(*I*^−^)]/[(*I*^+^)+(*I*^−^)] (Parsons *et al.*, 2013 ▸)
Absolute structure parameter	0.0 (10)

Computer programs: *APEX4* and *SAINT* (Bruker, 2021 ▸), *SHELXT* (Sheldrick, 2015*a* ▸), *SHELXL* (Sheldrick, 2015*b* ▸), *DIAMOND* (Brandenburg & Putz, 2012 ▸) and *SHELXTL* (Sheldrick, 2008 ▸).

## supplementary crystallographic information

### 2,5-Dimethyl-1H-benzo[d]imidazole Crystal data

**Table:**

C_9_H_10_N_2_	*D*_x_ = 1.227 Mg m^−^^3^
*M**_r_* = 146.19	Mo *K*α radiation, λ = 0.71073 Å
Orthorhombic, *P**n**a*2_1_	Cell parameters from 2151 reflections
*a* = 10.011 (3) Å	θ = 2.6–26.0°
*b* = 13.047 (4) Å	µ = 0.08 mm^−^^1^
*c* = 6.0600 (19) Å	*T* = 125 K
*V* = 791.5 (4) Å^3^	Plate, colourless
*Z* = 4	0.26 × 0.23 × 0.05 mm
*F*(000) = 312	

### 2,5-Dimethyl-1H-benzo[d]imidazole Data collection

**Table:**

Bruker D8 QUEST PHOTON 3 diffractometer	1599 independent reflections
Radiation source: fine-focus sealed tube	1106 reflections with *I* > 2σ(*I*)
Graphite monochromator	*R*_int_ = 0.062
Detector resolution: 7.3910 pixels mm^-1^	θ_max_ = 26.3°, θ_min_ = 2.6°
ω scans	*h* = −12→12
Absorption correction: numerical (*SADABS*;Krause *et al.*, 2015)	*k* = −16→15
*T*_min_ = 0.98, *T*_max_ = 1.00	*l* = −7→7
5699 measured reflections	

### 2,5-Dimethyl-1H-benzo[d]imidazole Refinement

**Table:**

Refinement on *F*^2^	Secondary atom site location: difference Fourier map
Least-squares matrix: full	Hydrogen site location: mixed
*R*[*F*^2^ > 2σ(*F*^2^)] = 0.069	H-atom parameters constrained
*wR*(*F*^2^) = 0.206	*w* = 1/[σ^2^(*F*_o_^2^) + (0.102*P*)^2^ + 0.2624*P*] where *P* = (*F*_o_^2^ + 2*F*_c_^2^)/3
*S* = 1.09	(Δ/σ)_max_ < 0.001
1599 reflections	Δρ_max_ = 0.16 e Å^−^^3^
134 parameters	Δρ_min_ = −0.31 e Å^−^^3^
54 restraints	Absolute structure: Flack *x* determined using 373 quotients [(*I*^+^)-(*I*^-^)]/[(*I*^+^)+(*I*^-^)] (Parsons *et al.*, 2013)
Primary atom site location: dual	Absolute structure parameter: 0.0 (10)

### 2,5-Dimethyl-1H-benzo[d]imidazole Special details

**Table:**

Experimental. The diffraction data were collected in three sets of 363 frames (0.5° width in ω) at φ = 0, 120 and 240°. A scan time of 80 sec/frame was used.
Geometry. All esds (except the esd in the dihedral angle between two l.s. planes) are estimated using the full covariance matrix. The cell esds are taken into account individually in the estimation of esds in distances, angles and torsion angles; correlations between esds in cell parameters are only used when they are defined by crystal symmetry. An approximate (isotropic) treatment of cell esds is used for estimating esds involving l.s. planes.
Refinement. Refinement of F^2^ against ALL reflections. The weighted R-factor wR and goodness of fit S are based on F^2^, conventional R-factors R are based on F, with F set to zero for negative F^2^. The threshold expression of F^2^ > 2sigma(F^2^) is used only for calculating R-factors(gt) etc. and is not relevant to the choice of reflections for refinement. R-factors based on F^2^ are statistically about twice as large as those based on F, and R- factors based on ALL data will be even larger. Hydrogen atoms were included as riding contributions in idealized positions with isotropic displacement parameters tied to those of the attached atoms. There is "whole molecule" disorder involving a rotation of the molecule about its long axis by 180° and a small translation perpendicular to this axis and in the plane of the molecule. The two orientations are in the ratio 0.849 (4)/0.151 (4) and were refined with restraints that their geometries be comparable.

### 2,5-Dimethyl-1H-benzo[d]imidazole Fractional atomic coordinates and isotropic or equivalent isotropic displacement parameters (Å^2^)

**Table:**

	*x*	*y*	*z*	*U*_iso_*/*U*_eq_	Occ. (<1)
N1	0.5960 (4)	0.2625 (4)	0.2831 (14)	0.0399 (12)	0.849 (4)
N2	0.8194 (4)	0.2688 (3)	0.2997 (15)	0.0361 (11)	0.849 (4)
H2	0.903259	0.257886	0.262991	0.043*	0.849 (4)
C1	0.7759 (5)	0.3254 (4)	0.4778 (15)	0.0377 (13)	0.849 (4)
C2	0.8377 (5)	0.3791 (5)	0.6458 (16)	0.0445 (14)	0.849 (4)
H2A	0.932415	0.382750	0.651882	0.053*	0.849 (4)
C3	0.7621 (7)	0.4274 (8)	0.804 (2)	0.0471 (15)	0.849 (4)
C4	0.6207 (6)	0.4219 (5)	0.7911 (16)	0.0474 (14)	0.849 (4)
H4	0.568129	0.455758	0.899353	0.057*	0.849 (4)
C5	0.5584 (6)	0.3687 (5)	0.6253 (16)	0.0485 (15)	0.849 (4)
H5	0.463720	0.364678	0.620103	0.058*	0.849 (4)
C6	0.6351 (5)	0.3208 (4)	0.4655 (15)	0.0371 (13)	0.849 (4)
C7	0.7095 (5)	0.2323 (4)	0.1895 (16)	0.0365 (13)	0.849 (4)
C8	0.8233 (7)	0.4886 (6)	0.9896 (18)	0.0632 (19)	0.849 (4)
H8A	0.769770	0.479704	1.123593	0.095*	0.849 (4)
H8B	0.914590	0.464447	1.016696	0.095*	0.849 (4)
H8C	0.825374	0.561251	0.949021	0.095*	0.849 (4)
C9	0.7184 (6)	0.1664 (6)	−0.0064 (18)	0.0479 (16)	0.849 (4)
H9A	0.636070	0.126263	−0.020666	0.072*	0.849 (4)
H9B	0.730302	0.208950	−0.138196	0.072*	0.849 (4)
H9C	0.794705	0.119887	0.008945	0.072*	0.849 (4)
N1A	0.8890 (16)	0.2654 (19)	0.300 (3)	0.0399 (12)	0.151 (4)
N2A	0.6658 (15)	0.2699 (18)	0.327 (3)	0.0361 (11)	0.151 (4)
H2B	0.581480	0.258024	0.294358	0.043*	0.151 (4)
C1A	0.7116 (13)	0.327 (2)	0.502 (4)	0.0377 (13)	0.151 (4)
C2A	0.6526 (15)	0.382 (2)	0.671 (3)	0.0445 (14)	0.151 (4)
H2C	0.558058	0.386311	0.680323	0.053*	0.151 (4)
C3A	0.731 (2)	0.431 (5)	0.825 (7)	0.0471 (15)	0.151 (4)
C4A	0.8718 (18)	0.423 (2)	0.810 (4)	0.0474 (14)	0.151 (4)
H4A	0.926204	0.459690	0.911426	0.057*	0.151 (4)
C5A	0.9307 (15)	0.363 (2)	0.653 (3)	0.0485 (15)	0.151 (4)
H5A	1.024173	0.349871	0.656622	0.058*	0.151 (4)
C6A	0.8522 (14)	0.320 (2)	0.488 (3)	0.0371 (13)	0.151 (4)
C7A	0.7743 (17)	0.235 (2)	0.211 (4)	0.0365 (13)	0.151 (4)
C8A	0.672 (2)	0.490 (3)	1.017 (4)	0.0632 (19)	0.151 (4)
H8D	0.699138	0.456659	1.155459	0.095*	0.151 (4)
H8E	0.704925	0.560273	1.014462	0.095*	0.151 (4)
H8F	0.574402	0.489558	1.006254	0.095*	0.151 (4)
C9A	0.763 (3)	0.176 (4)	0.005 (6)	0.0479 (16)	0.151 (4)
H9D	0.734716	0.105956	0.038208	0.072*	0.151 (4)
H9E	0.697297	0.208692	−0.092148	0.072*	0.151 (4)
H9F	0.850182	0.174415	−0.069640	0.072*	0.151 (4)

### 2,5-Dimethyl-1H-benzo[d]imidazole Atomic displacement parameters (Å^2^)

**Table:**

	*U*^11^	*U*^22^	*U*^33^	*U*^12^	*U*^13^	*U*^23^
N1	0.028 (2)	0.058 (3)	0.033 (2)	0.004 (2)	0.009 (3)	0.001 (2)
N2	0.0151 (19)	0.055 (3)	0.038 (2)	0.0013 (17)	0.010 (2)	0.007 (2)
C1	0.035 (3)	0.043 (3)	0.036 (3)	0.002 (3)	−0.004 (3)	0.008 (2)
C2	0.040 (3)	0.049 (3)	0.045 (3)	−0.007 (3)	−0.004 (3)	0.010 (3)
C3	0.058 (4)	0.047 (3)	0.037 (3)	0.001 (4)	−0.003 (3)	0.003 (3)
C4	0.054 (3)	0.049 (3)	0.039 (3)	0.005 (3)	0.007 (3)	0.003 (3)
C5	0.041 (3)	0.065 (4)	0.039 (3)	−0.001 (3)	0.007 (3)	0.000 (3)
C6	0.034 (3)	0.044 (3)	0.033 (3)	0.004 (2)	0.004 (3)	0.009 (3)
C7	0.028 (3)	0.050 (3)	0.032 (2)	−0.001 (3)	0.007 (3)	0.009 (2)
C8	0.082 (5)	0.061 (4)	0.046 (4)	−0.011 (3)	−0.009 (4)	0.001 (3)
C9	0.044 (4)	0.061 (4)	0.039 (3)	−0.003 (4)	0.012 (4)	0.000 (3)
N1A	0.028 (2)	0.058 (3)	0.033 (2)	0.004 (2)	0.009 (3)	0.001 (2)
N2A	0.0151 (19)	0.055 (3)	0.038 (2)	0.0013 (17)	0.010 (2)	0.007 (2)
C1A	0.035 (3)	0.043 (3)	0.036 (3)	0.002 (3)	−0.004 (3)	0.008 (2)
C2A	0.040 (3)	0.049 (3)	0.045 (3)	−0.007 (3)	−0.004 (3)	0.010 (3)
C3A	0.058 (4)	0.047 (3)	0.037 (3)	0.001 (4)	−0.003 (3)	0.003 (3)
C4A	0.054 (3)	0.049 (3)	0.039 (3)	0.005 (3)	0.007 (3)	0.003 (3)
C5A	0.041 (3)	0.065 (4)	0.039 (3)	−0.001 (3)	0.007 (3)	0.000 (3)
C6A	0.034 (3)	0.044 (3)	0.033 (3)	0.004 (2)	0.004 (3)	0.009 (3)
C7A	0.028 (3)	0.050 (3)	0.032 (2)	−0.001 (3)	0.007 (3)	0.009 (2)
C8A	0.082 (5)	0.061 (4)	0.046 (4)	−0.011 (3)	−0.009 (4)	0.001 (3)
C9A	0.044 (4)	0.061 (4)	0.039 (3)	−0.003 (4)	0.012 (4)	0.000 (3)

### 2,5-Dimethyl-1H-benzo[d]imidazole Geometric parameters (Å, º)

**Table:**

N1—C7	1.330 (6)	N1A—C7A	1.330 (7)
N1—C6	1.398 (8)	N1A—C6A	1.397 (8)
N2—C7	1.372 (6)	N2A—C7A	1.372 (7)
N2—C1	1.378 (8)	N2A—C1A	1.379 (8)
N2—H2	0.8800	N2A—H2B	0.8800
C1—C2	1.383 (8)	C1A—C2A	1.383 (9)
C1—C6	1.413 (8)	C1A—C6A	1.413 (8)
C2—C3	1.375 (9)	C2A—C3A	1.375 (9)
C2—H2A	0.9500	C2A—H2C	0.9500
C3—C4	1.419 (9)	C3A—C4A	1.420 (9)
C3—C8	1.509 (10)	C3A—C8A	1.509 (10)
C4—C5	1.371 (9)	C4A—C5A	1.371 (9)
C4—H4	0.9500	C4A—H4A	0.9500
C5—C6	1.385 (8)	C5A—C6A	1.385 (8)
C5—H5	0.9500	C5A—H5A	0.9500
C7—C9	1.469 (8)	C7A—C9A	1.469 (9)
C8—H8A	0.9800	C8A—H8D	0.9800
C8—H8B	0.9800	C8A—H8E	0.9800
C8—H8C	0.9800	C8A—H8F	0.9800
C9—H9A	0.9800	C9A—H9D	0.9800
C9—H9B	0.9800	C9A—H9E	0.9800
C9—H9C	0.9800	C9A—H9F	0.9800
			
C7—N1—C6	105.0 (4)	C7A—N1A—C6A	104.9 (5)
C7—N2—C1	108.3 (4)	C7A—N2A—C1A	108.2 (5)
C7—N2—H2	125.9	C7A—N2A—H2B	125.9
C1—N2—H2	125.9	C1A—N2A—H2B	125.9
N2—C1—C2	135.0 (5)	N2A—C1A—C2A	135.3 (6)
N2—C1—C6	104.5 (5)	N2A—C1A—C6A	104.5 (6)
C2—C1—C6	120.5 (5)	C2A—C1A—C6A	120.2 (6)
C3—C2—C1	120.0 (5)	C3A—C2A—C1A	120.1 (6)
C3—C2—H2A	120.0	C3A—C2A—H2C	119.9
C1—C2—H2A	120.0	C1A—C2A—H2C	119.9
C2—C3—C4	119.2 (6)	C2A—C3A—C4A	119.1 (7)
C2—C3—C8	122.5 (6)	C2A—C3A—C8A	122.5 (8)
C4—C3—C8	118.3 (6)	C4A—C3A—C8A	118.2 (8)
C5—C4—C3	121.3 (6)	C5A—C4A—C3A	121.1 (7)
C5—C4—H4	119.3	C5A—C4A—H4A	119.5
C3—C4—H4	119.3	C3A—C4A—H4A	119.5
C4—C5—C6	119.3 (5)	C4A—C5A—C6A	119.1 (7)
C4—C5—H5	120.4	C4A—C5A—H5A	120.5
C6—C5—H5	120.4	C6A—C5A—H5A	120.5
C5—C6—N1	130.0 (5)	C5A—C6A—N1A	130.1 (6)
C5—C6—C1	119.8 (5)	C5A—C6A—C1A	119.8 (6)
N1—C6—C1	110.2 (5)	N1A—C6A—C1A	110.1 (5)
N1—C7—N2	112.0 (5)	N1A—C7A—N2A	112.0 (5)
N1—C7—C9	124.7 (5)	N1A—C7A—C9A	124.5 (6)
N2—C7—C9	123.2 (5)	N2A—C7A—C9A	123.4 (6)
C3—C8—H8A	109.4	C3A—C8A—H8D	109.5
C3—C8—H8B	109.5	C3A—C8A—H8E	109.3
H8A—C8—H8B	109.5	H8D—C8A—H8E	109.5
C3—C8—H8C	109.5	C3A—C8A—H8F	109.6
H8A—C8—H8C	109.5	H8D—C8A—H8F	109.5
H8B—C8—H8C	109.5	H8E—C8A—H8F	109.5
C7—C9—H9A	109.5	C7A—C9A—H9D	109.3
C7—C9—H9B	109.5	C7A—C9A—H9E	109.5
H9A—C9—H9B	109.5	H9D—C9A—H9E	109.5
C7—C9—H9C	109.4	C7A—C9A—H9F	109.6
H9A—C9—H9C	109.5	H9D—C9A—H9F	109.5
H9B—C9—H9C	109.5	H9E—C9A—H9F	109.5
			
C7—N2—C1—C2	179.6 (7)	C7A—N2A—C1A—C2A	179 (4)
C7—N2—C1—C6	−0.4 (6)	C7A—N2A—C1A—C6A	−2 (4)
N2—C1—C2—C3	−179.0 (8)	N2A—C1A—C2A—C3A	179 (5)
C6—C1—C2—C3	0.9 (11)	C6A—C1A—C2A—C3A	1 (6)
C1—C2—C3—C4	−0.6 (14)	C1A—C2A—C3A—C4A	−1 (8)
C1—C2—C3—C8	−179.1 (8)	C1A—C2A—C3A—C8A	−178 (4)
C2—C3—C4—C5	0.7 (14)	C2A—C3A—C4A—C5A	−3 (7)
C8—C3—C4—C5	179.2 (7)	C8A—C3A—C4A—C5A	173 (4)
C3—C4—C5—C6	−1.1 (11)	C3A—C4A—C5A—C6A	9 (5)
C4—C5—C6—N1	179.7 (6)	C4A—C5A—C6A—N1A	173 (3)
C4—C5—C6—C1	1.4 (9)	C4A—C5A—C6A—C1A	−9 (4)
C7—N1—C6—C5	−178.0 (6)	C7A—N1A—C6A—C5A	174 (3)
C7—N1—C6—C1	0.4 (6)	C7A—N1A—C6A—C1A	−4 (3)
N2—C1—C6—C5	178.6 (5)	N2A—C1A—C6A—C5A	−174 (3)
C2—C1—C6—C5	−1.3 (9)	C2A—C1A—C6A—C5A	4 (5)
N2—C1—C6—N1	0.0 (6)	N2A—C1A—C6A—N1A	4 (4)
C2—C1—C6—N1	−180.0 (5)	C2A—C1A—C6A—N1A	−177 (3)
C6—N1—C7—N2	−0.7 (6)	C6A—N1A—C7A—N2A	2 (4)
C6—N1—C7—C9	178.4 (6)	C6A—N1A—C7A—C9A	178 (4)
C1—N2—C7—N1	0.7 (6)	C1A—N2A—C7A—N1A	0 (4)
C1—N2—C7—C9	−178.4 (6)	C1A—N2A—C7A—C9A	−176 (4)

### 2,5-Dimethyl-1H-benzo[d]imidazole Hydrogen-bond geometry (Å, º)

*Cg*1 and *Cg*2 are the centroids of the N1/C6/C1/N2/C7 and C1–C6 rings, respectively.

**Table:**

*D*—H···*A*	*D*—H	H···*A*	*D*···*A*	*D*—H···*A*
N2—H2···N1^i^	0.88	1.95	2.801 (6)	162
C8—H8*C*···Cg1^ii^	0.98	2.97	3.941 (8)	172
C9—H9*B*···Cg2^iii^	0.98	2.57	3.478 (10)	153

Symmetry codes: (i) *x*+1/2, −*y*+1/2, *z*; (ii) *x*+3/2, −*y*+1/2, *z*; (iii) *x*, *y*, *z*−1.
